# Transporting an Artificial Intelligence Model to Predict Emergency Cesarean Delivery: Overcoming Challenges Posed by Interfacility Variation

**DOI:** 10.2196/28120

**Published:** 2021-12-10

**Authors:** Joshua Guedalia, Michal Lipschuetz, Sarah M Cohen, Yishai Sompolinsky, Asnat Walfisch, Eyal Sheiner, Ruslan Sergienko, Joshua Rosenbloom, Ron Unger, Simcha Yagel, Hila Hochler

**Affiliations:** 1 The Mina and Everard Goodman Faculty of Life Sciences Bar-Ilan University Ramat Gan Israel; 2 Division of Obstetrics & Gynecology Hadassah Medical Organization and Faculty of Medicine Hebrew University of Jerusalem Jerusalem Israel; 3 Department of Obstetrics and Gynecology Soroka University Medical Center Ben-Gurion University of the Negev Beer-Sheva Israel; 4 Department of Public Health School of Public Health, Faculty of Health Sciences Ben-Gurion University of the Negev Beer-Sheva Israel

**Keywords:** machine learning, algorithm transport, health outcomes, health care facilities, artificial intelligence, AI, ML, pregnancy, birth, pediatrics, neonatal, prenatal

## Abstract

Research using artificial intelligence (AI) in medicine is expected to significantly influence the practice of medicine and the delivery of health care in the near future. However, for successful deployment, the results must be transported across health care facilities. We present a cross-facilities application of an AI model that predicts the need for an emergency caesarean during birth. The transported model showed benefit; however, there can be challenges associated with interfacility variation in reporting practices.

## Introduction

The integration of artificial intelligence (AI) into health care is expected to significantly influence the practice of medicine [1-4]. Machine learning (ML) as a modeling strategy is an attractive option for characterizing and predicting complex biological phenomena [5].

Critics of AI applications note that the applications are primarily based on retrospective research, with insufficient focus devoted to “real-life” implementation and verification of reproducibility in clinical practice [5,6]. For example, an ML prediction algorithm developed in an urban tertiary care center with a diverse patient population may be unsuitable for a community hospital treating a homogenous population according to local protocols.

Therefore, transporting AI models across health care facilities is critical to effectively translating AI research into medical practice [7]. In this study, we aimed to investigate the validation of a model to predict the need for an emergency caesarean during birth, the critical challenge stemming from interfacility variation in subjective measurements, and to devise a method to address this challenge.

## Methods

In brief, we developed 2 ML models to predict the risk for emergency caesarean delivery (for a detailed description of the methods and model features, see Multimedia Appendix 1 and [8]). The first model was designed to be used at admission to the labor and delivery unit (admission model); the second model was designed for use during labor, integrating additional data that accumulate as labor progresses (labor progression model). These additional data supplementing the model allow for more accurate prediction. Both models will alert the staff of the likelihood that a parturient might require an emergency caesarean delivery, allowing for the preparation of staff and patient.

The models were trained using data from approximately 100,000 births at Hospital A. We extracted multiple data features from individual parturient electronic medical records (EMRs), totaling approximately 11 million data points. The institutional review boards at Hadassah Hebrew University Medical Center and Soroka Medical Center approved the study.

Both models were able to predict the need for emergency caesarean delivery, with the admission model achieving an area under the curve (AUC) of 0.82 and the labor progression model showing an increased performance, with an AUC of 0.86.

Having created and trained an ML-based model at a given health care facility, model transport can provide a smaller facility its benefits, without the large stored medical records or the expense and expertise required for development. However, care must be taken to monitor how the transport may affect the performance of the models, given differences in populations or settings.

**Figure 1:**
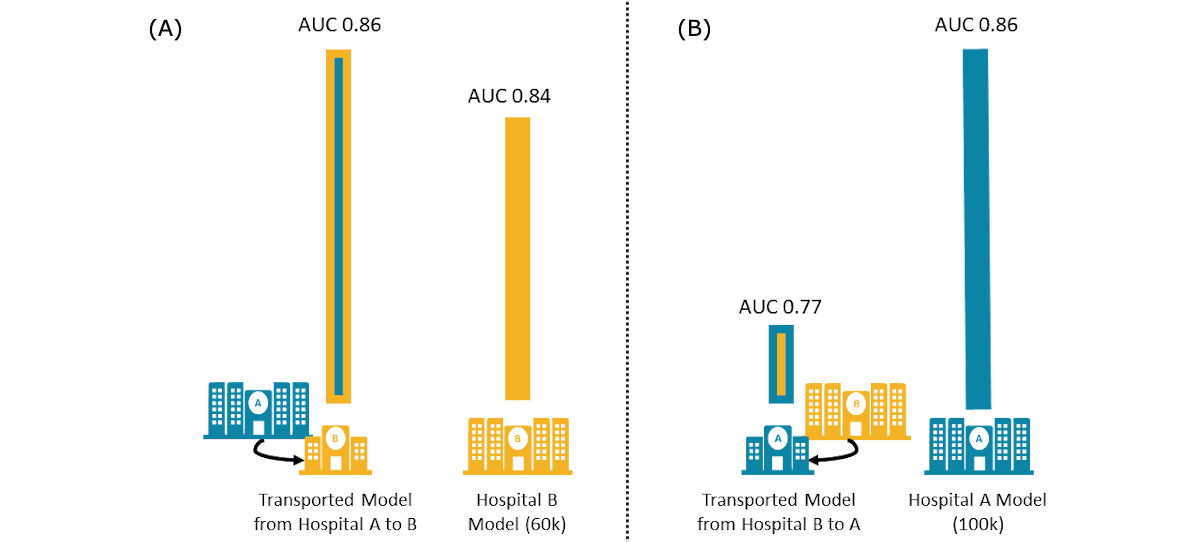
(A) Comparing the performance of Hospital A labor progression model (in blue) transported to Hospital B (yellow/blue bar) versus Hospital B local model (in yellow) and (B) Comparing the performance of Hospital B labor progression model (in yellow) transported to Hospital A (blue/yellow bar) versus Hospital A local model (in blue). AUC: area under the curve.

We compared the prediction performance of the models trained and tested at Hospital A when transported to a second facility, Hospital B, where they were tested on data from approximately 60,000 births. Both the admission and labor progression models transported from Hospital A showed comparable prediction performance at Hospital B. Figure 1A illustrates the transport and performance of the labor progression model (see Multimedia Appendix 2 for the hospital characteristics and Multimedia Appendix 3 for the AUCs and 95% CIs of all models).

We then reversed the process and retested the success of transporting the models, by training the models at Hospital B and testing the prediction accuracy at Hospital A. Although the admission model trained at Hospital B provided similar levels of prediction at Hospital A, the labor progression model showed a reduced level of prediction (AUC 0.77 vs AUC 0.84; Figure 1B). We examined the model features to determine the cause of this decreased performance (see Multimedia Appendix 1).

Two important measurements of labor progression are fetal head station and cervical dilation. Fetal head station denotes the fetal descent within the maternal pelvis based on the position of the fetal head in centimeters above (–) or below (+) the maternal ischial spines [9]. Cervical dilation refers to the opening of the maternal uterine cervix, in centimeters, from closed cervix (0 cm) to full cervical dilation (10 cm). These 2 measurements represent the primary features of the progress of the birth; how rapidly descent and dilation progress depends on several factors, including parturient parity, medical history, pelvic anatomy, the size of the fetus, and the position of the fetus at the time of labor [10]. Results are operator-dependent, and measurements can vary between facilities based on local protocols and practice habits [11].

We identified a difference between the 2 facilities in fetal head station measurements used by the labor progression model. Specifically, we found that the dispersion and central tendency of this variable, as stratified to cervical dilation, differed between the 2 hospitals: Data from Hospital A were widely distributed across the full –3 to +3 scale, while those from Hospital B were more concentrated around –2 to +2. This difference may explain the reduced performance when transporting from Hospital B, while no reduction in performance was observed when transporting from Hospital A.

**Figure 2:**
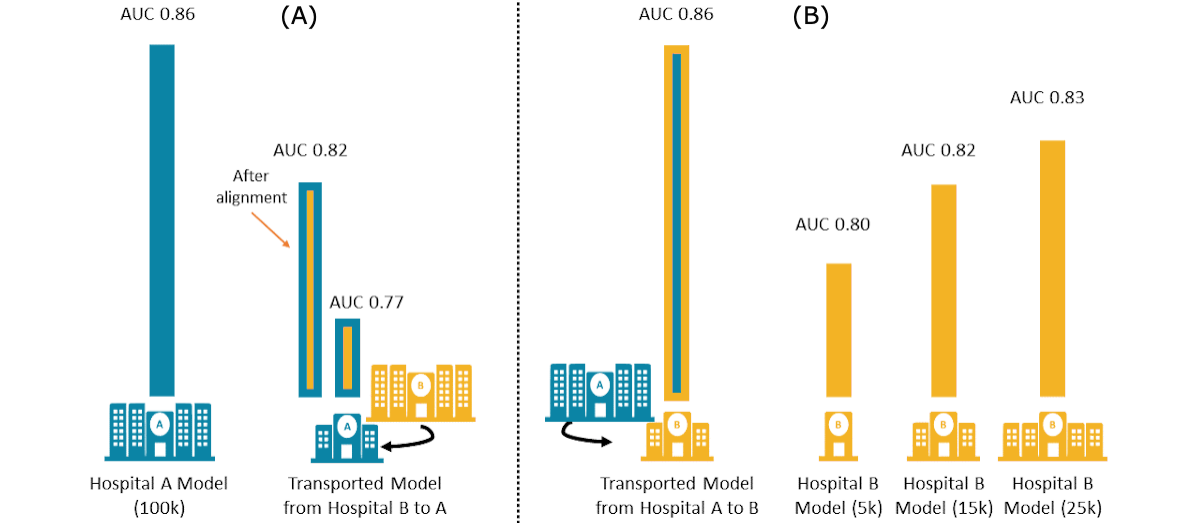
(A) Comparing the performance of Hospital B labor progression model (in yellow) transported to Hospital A versus Hospital B model after alignment adjustments transported to Hospital A (blue/yellow bars) versus Hospital A local model (in blue) and (B) Comparing the performance of Hospital A labor progression model transported to Hospital B (yellow/blue bar) versus Hospital B local models trained on progressively larger local electronic medical record (EMR) data sets of 5000, 15,000, and 25,000 (in yellow). AUC: area under the curve.

In order to overcome this disparity, we evaluated the patterns of distribution of fetal head station as distributed across the dilation. We aligned the station within the distribution of the cervical dilation in order to encompass both approaches. This partly adjusted for the variation and improved the cross-facility prediction (AUC 0.82; Figure 2A; see Multimedia Appendix 1 and Multimedia Appendix 3 for the AUCs and 95% CIs of all models).

This difference highlights the difficulties introduced by discrepancies in reporting practices between facilities. Alignment can resolve some disparities, but here, it only partly recouped model performance.

To further evaluate whether our labor progression model could potentially benefit an even smaller facility, we simulated a hospital with a smaller EMR. The 100,000-case Hospital A model transported to Hospital B showed better performance (AUC 0.86) than a Hospital B model based on small samples of 5000 (AUC 0.80), 15,000 (AUC 0.82), and 25,000 (AUC 0.83) cases, emphasizing the benefit that can accrue to a smaller facility from a model trained at a larger facility and that the additional benefit decreases as the size of the available local EMR grows (Figure 2B).

### Conclusions

In conclusion, integrating ML applications into clinical medicine will require validation and transportation between medical facilities [7,12-14]. We demonstrated that ML may be applied to clinical practice and to obstetrics in particular. A big data–driven ML algorithm can be successfully transported, and a data-poor center can benefit from work performed in a larger facility.

However, transportation requires careful investigation of specific features and consideration of variations in local populations, protocols, and reporting to calibrate the system fit [7,12]. Nevertheless, model predictions are heavily dependent on the data used in training and by the variations in recording practices and protocols operative in a given health care facility. We observed that the more detailed labor progression model, when trained without accounting for reporting differences, provided a lower AUC than the admission model. Although the progression model contained more detailed information on the progression of the labor and intrahospital showed benefit over the admission model, the benefit provided was lost when transporting the model to a different hospital: The transported model performance was inferior to that of the simpler model. Interfacility variation between health care centers may introduce unexpected effects into a prediction model. Generalizability and transportability among medical facilities necessitate overcoming biases via external validation and adapting the model to local protocols [15].

Successful translation of AI research into practice depends on transport across health care facilities. This can individualize health care, improve outcomes, and reduce complications across broader populations.

## Appendix

Multimedia Appendix 1 Additional methodology.

Multimedia Appendix 2 Demographic parameters of the two hospitals.

Multimedia Appendix 3 AUROC of the different models.

## Abbreviations

AI: artificial intelligence
AUC: area under the curve
EMR: electronic medical record
ML: machine learning
