# Supplementary material for: Transporting an Artificial Intelligence Model to Predict Emergency Cesarean Delivery: Overcoming Challenges Posed by Interfacility Variation
Source: J Med Internet Res. 2021 Dec 10;23(12):e28120. doi: 10.2196/28120 (PMC8709908; doi:10.2196/28120)
Supplement: Multimedia Appendix 1 [file jmir_v23i12e28120_app1.docx]

**Multimedia Appendix 1.** Additional methodology.

*Creation of the prediction models*

We previously conducted a retrospective database analysis of some 100,000 trials of labor at a tertiary medical center in Israel, to create prediction models for successful vaginal delivery in singleton births [1].

Multiple data features covering maternal demographic and obstetric history, gestational age, labor and delivery course and outcomes were collected. Data on labor progression included repeated, time-stamped, cervical dilation and fetal-head station measurements.

Analysis was performed with Python 3.7.3, scikit‐learn library 0.21.2, catboost 0.15.2, Office Excel 2010 (Microsoft, Seattle, WA). Gradient boosting machine learning was used to train the prediction models. This method builds a model using a collection of decision trees based on training data, where each consecutive tree is trained with focus on the previous trees’ errors. We selected CatBoost implementation of gradient boosting [2]. The model was then used to predict outcomes by combining the predictions of the ensemble of decision trees. Predication accuracy was measured by the area under the curve (AUC) of the receiver operating characteristic (ROC) curve.

We trained models using data available at two timepoints: on admission to the delivery unit (admission model), and up to the end of the first stage of labor, at cervical dilation of 10cm (labor progression model). Parturients that were delivered via cesarean prior to reaching the end of the first stage were excluded from the labor progression model.

Aggregation of all the data available at the end of the first stage was performed and provided as additional features to the models. The aggregation calculates all data records up until that point for each feature including count, mean, minimum, maximum, standard deviation, first recorded value, last recorded value, and delta between maximum and minimum recordings. This aggregation method enables progression and trend analysis during labor.

Median imputation, a common approach to dealing with missing values in machine-learning algorithms [3], was applied in this study to the data acquired for the admission model. This enables analysis of the whole cohort, as opposed to analysis of only a subset of the cohort with complete datasets.

*Transportation of the models*

In the present study we evaluated transportation of models across hospitals. The study was performed at the two campuses of the Hadassah Hebrew University Medical Center in Jerusalem (Hospital A) and Soroka University Medical Center in Beer Sheba (Hospital B). Each model requires data for training and testing, while the testing data must be ‘put aside’ and not used during training. We allocated 20% of the data for testing, i.e., the ‘test set’. In order to validate the results, we ran a 10-fold test, running all tests 10 times, each time with a different, randomly stratified sampled test set.

We then created the following model variations:

1. **Local model at Hospital A** - Train a model at Hospital A and test it on Hospital A’s test set.
2. **External model transported from Hospital B to Hospital A** - Train a model at Hospital B and test it on Hospital A’s test set.
3. **Simulated small EMR** **at Hospital A** - Train a model at Hospital A using only a sample of the data available and test it on Hospital A’s test set.
   We sampled at 5,000 EMR intervals (eg, 5k, 10k, 15k, etc.). We sampled 4 times, each time with a different randomly stratified sample.

The AUCs for all 10 times we ran the tests, and for the 4 times we sampled the simulated small EMR, were then averaged across all the tests.

We evaluated both the admission and progression of labor models on successively larger simulated data sets (5k, 10k, etc.), for each hospital against the other, and then reversed the process. This generated approximately 40 models. Review of the AUCs of each model, revealed that the admission model saw a performance plateau earlier than the progression of labor model. The progression model is a more detailed model (ie, it contains more features).

Note: when comparing models, we compared the results against the same test set (see Supplemental Figure 1).

**Supplmental Figure 1.** Schematic description of evaluating the need of transformation of a model from an external Hospital (A- blue to yellow) vs. local model (B- in yellow). Both models are tested against the same local test set (B), then a comparison is performed between the two models to evaluate which is the superior model.


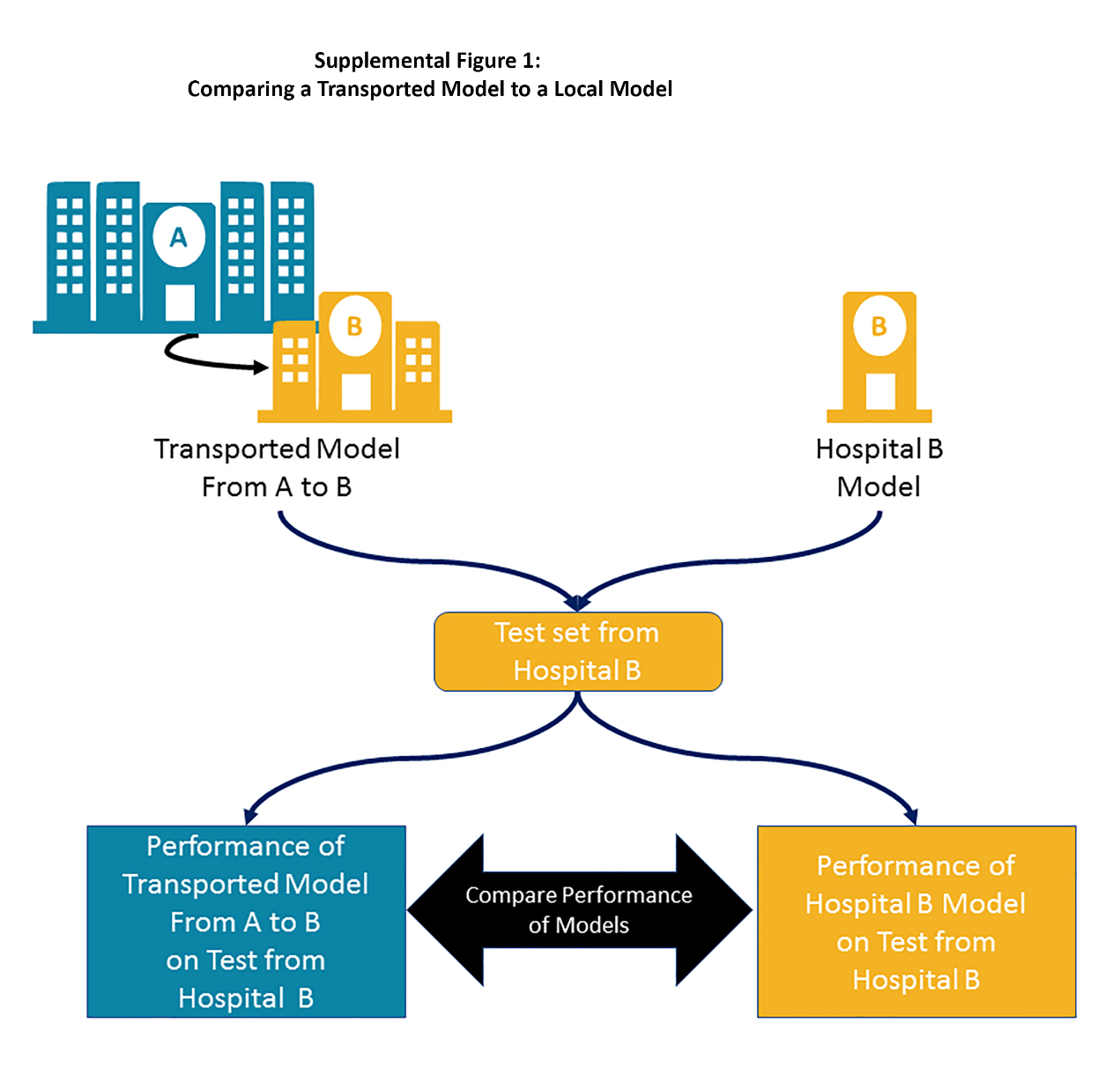


This process was repeated for each hospital and for each model (admission and labor progression). In order to evaluate the transport of the models we reversed the order of hospitals, switching Hospital A with Hospital B, and ran the tests again.

*Identifying the cause of decreased performance*

We created a ML model to attempt to classify from which hospital the data originated based on the same features used to classify the delivery mode. This model highlighted the fetal head station as of primary importance.

*Alignment*

We evaluated the features of fetal head station and cervical dilation in each of the two hospitals. Fetal head station expresses the fetal head decent in the maternal pelvis in centimeters, relative to the level of the maternal ischial spines, while cervical dilation refers to the opening of the maternal uterine cervix, in centimeters, with 10 cm denoting full dilation. Analysis showed that the measurements differed in dispersion and central tendency: those from Hospital A were distributed more widely across the possible scale, while the measurements from Hospital B were more concentrated toward the center. Based on these distinctive distributions of fetal head station, mapped against those of cervical dilation recorded within each hospital’s dataset, we aligned the distribution of fetal head station in order to encompass the centers’ varying approaches.

We attempted to align the features via centering and scaling, as well as by removing the extremities of the scales: neither action improved the accuracy of cross facility models. In order to align the two hospitals’ scales we used expert clinical domain knowledge to ascertain the dependence between two features, fetal head station and cervical dilation. They are multidimensional features that represent interdependent clinical examinations.

Comparing each of the hospitals’ head station measurements and cervical dilation distribution to each other, we revealed varying dilation distribution. We then aligned the station scales such that the dilation distribution within each station measurement would match.

**References**

1. Guedalia J, Lipschuetz M, Novoselsky-Persky M, Cohen SM, Rottenstreich A, Levin G, et al. Real-time data analysis using a machine learning model significantly improves prediction of successful vaginal deliveries. Am J Obstet Gynecol. 2020 Sep;223(3):437 e1- e15. PMID: 32434000
2. Prokhorenkova L, Gusev G, Vorobev A, Dorogush AV, Gulin A. CatBoost: unbiased boosting with categorical features. arXiv preprint arXiv:170609516. 2017. <https://arxiv.org/abs/1706.09516>
3. Batista GE, Monard MC. An analysis of four missing data treatment methods for supervised learning. Applied artificial intelligence. 2003;17(5-6):519-33.
