# Supplementary material for: Transporting an Artificial Intelligence Model to Predict Emergency Cesarean Delivery: Overcoming Challenges Posed by Interfacility Variation
Source: J Med Internet Res. 2021 Dec 10;23(12):e28120. doi: 10.2196/28120 (PMC8709908; doi:10.2196/28120)
Supplement: Multimedia Appendix 2 [file jmir_v23i12e28120_app2.docx]

**Multimedia Appendix 2.** Demographic parameters of the two hospitals.

|  | **Hospital 1 (n=61,012)** | **Hospital 2 (n=102,433)** | **P value*** |
| --- | --- | --- | --- |
| **Maternal age (yrs.) (median, IQR)** | 28.0 (24.0-33.0) | 29.0 (25.0-33.0) | <0.001 |
| **Primipara (first delivery) (%)** | 15,981 (26.2%) | 28,592 (27.9%) | <0.001 |
| **Gestational age (median, IQR)** | 39.9 (39.0-40.6) | 40.0 (39.2-40.8) | <0.001 |
| **Previous cesarean delivery (%)** | 5,326 (8.7%) | 8,325 (8.1%) | <0.001 |
| **Induction of labor (%)** | 5,644 (9.3%) | 16,164 (15.8%) | <0.001 |
| **Delivery mode: (%)** |  |  |  |
| **Vaginal** | 55,899 (91.6%) | 89,420 (87.3%) | <0.001 |
| **Instrumental** | 2,282 (3.7%) | 7,802 (7.6%) |  |
| **Unplanned CD** | 2,831 (4.6%) | 5,211 (5.1%) |  |

*Chi square or Mann-Whitney U
