# Supplementary material for: Transporting an Artificial Intelligence Model to Predict Emergency Cesarean Delivery: Overcoming Challenges Posed by Interfacility Variation
Source: J Med Internet Res. 2021 Dec 10;23(12):e28120. doi: 10.2196/28120 (PMC8709908; doi:10.2196/28120)
Supplement: Multimedia Appendix 3 [file jmir_v23i12e28120_app3.docx]

**Multimedia Appendix 3.** AUROC of the different models.

| **Model description** | **Figure** | **AUC (95% CI)** |
| --- | --- | --- |
| Transported Model  from Hospital A to B | figure 1a1 | 0.86 (0.83-0.89) |
| Hospital B  Model (60k) | figure 1a2 | 0.84 (0.80-0.87) |
| Transported Model  from Hospital B to A | figure 1b1 | 0.77 (0.74-0.80) |
| Hospital A Model  (100k) | figure 1b2 | 0.86 (0.84-0.88) |
| Hospital A Model  (100k) | figure 2a1 | 0.86 (0.84-0.88) |
| Transported Model  from Hospital B to A – After Alignment | figure 2a2 | 0.82 (0.80-0.84) |
| Transported Model  from Hospital B to A | figure 2a3 | 0.77 (0.74-0.80) |
| Transported Model  from Hospital A to B | figure 2b1 | 0.86 (0.83-0.89) |
| Hospital B  Model (5k) | figure 2b2 | 0.80 (0.76-0.83) |
| Hospital B  Model (15k) | figure 2b3 | 0.82 (0.79-0.86) |
| Hospital B  Model (25k) | figure 2b4 | 0.83 (0.80-0.87) |
